# Supplementary figures and images for: Structural characterization of the full-length Hantaan virus polymerase
Source: PLoS Pathog. 2024 Dec 9;20(12):e1012781. doi: 10.1371/journal.ppat.1012781 (PMC11658695; doi:10.1371/journal.ppat.1012781)

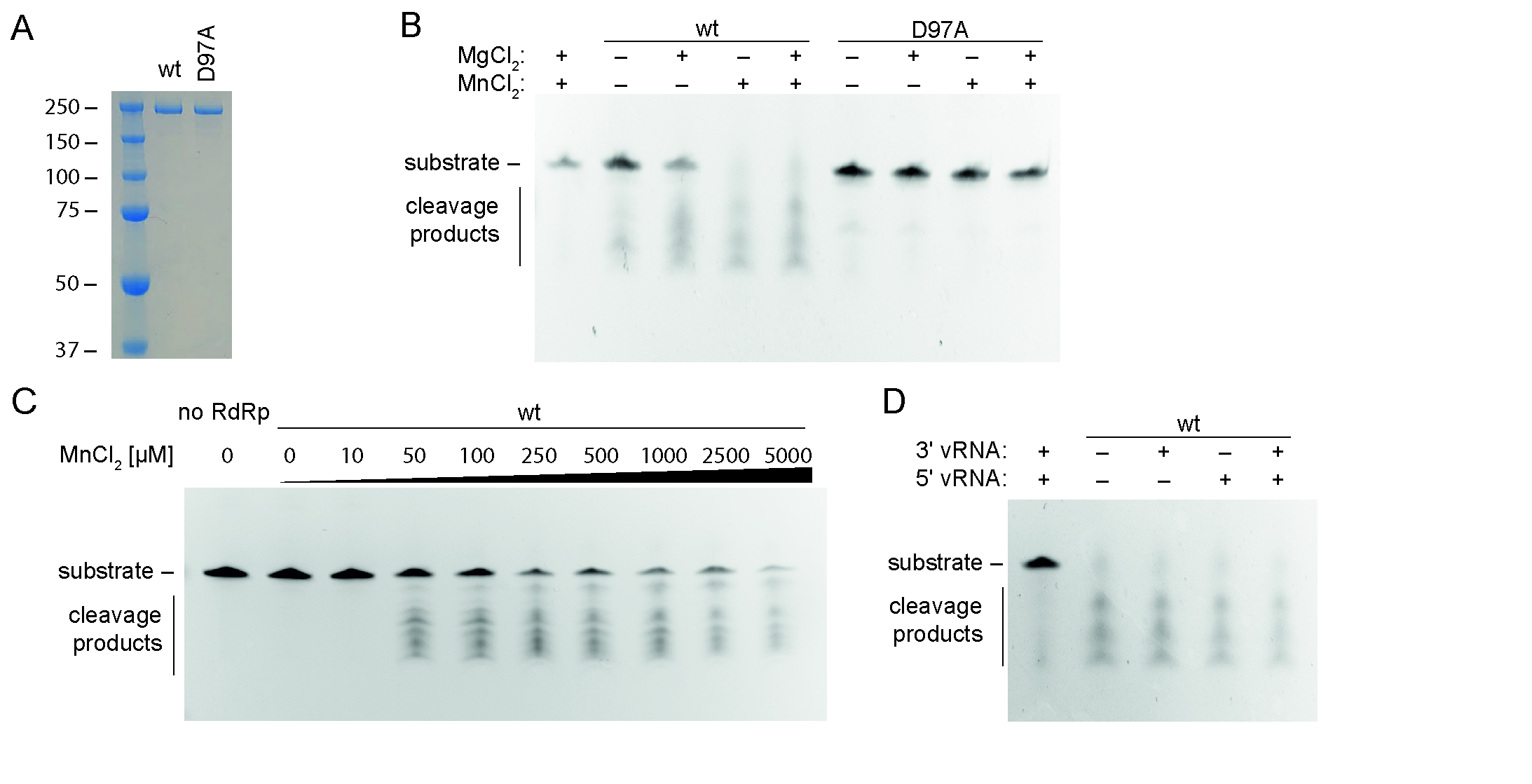

Supplement: S1 Fig — (A) SDS-page analysis of the wt and D97A HTNV viral polymerase preparations. (B) Endonuclease activity assays in the presence or absence of divalent cations at fixed concentrations alone or in combination. (C) Titration of MnCl2 to determine the effect on endonuclease activity. (D) Addition of viral RNA promoters to the endonuclease assay did not affect activity. (TIF) [file ppat.1012781.s002.tif]

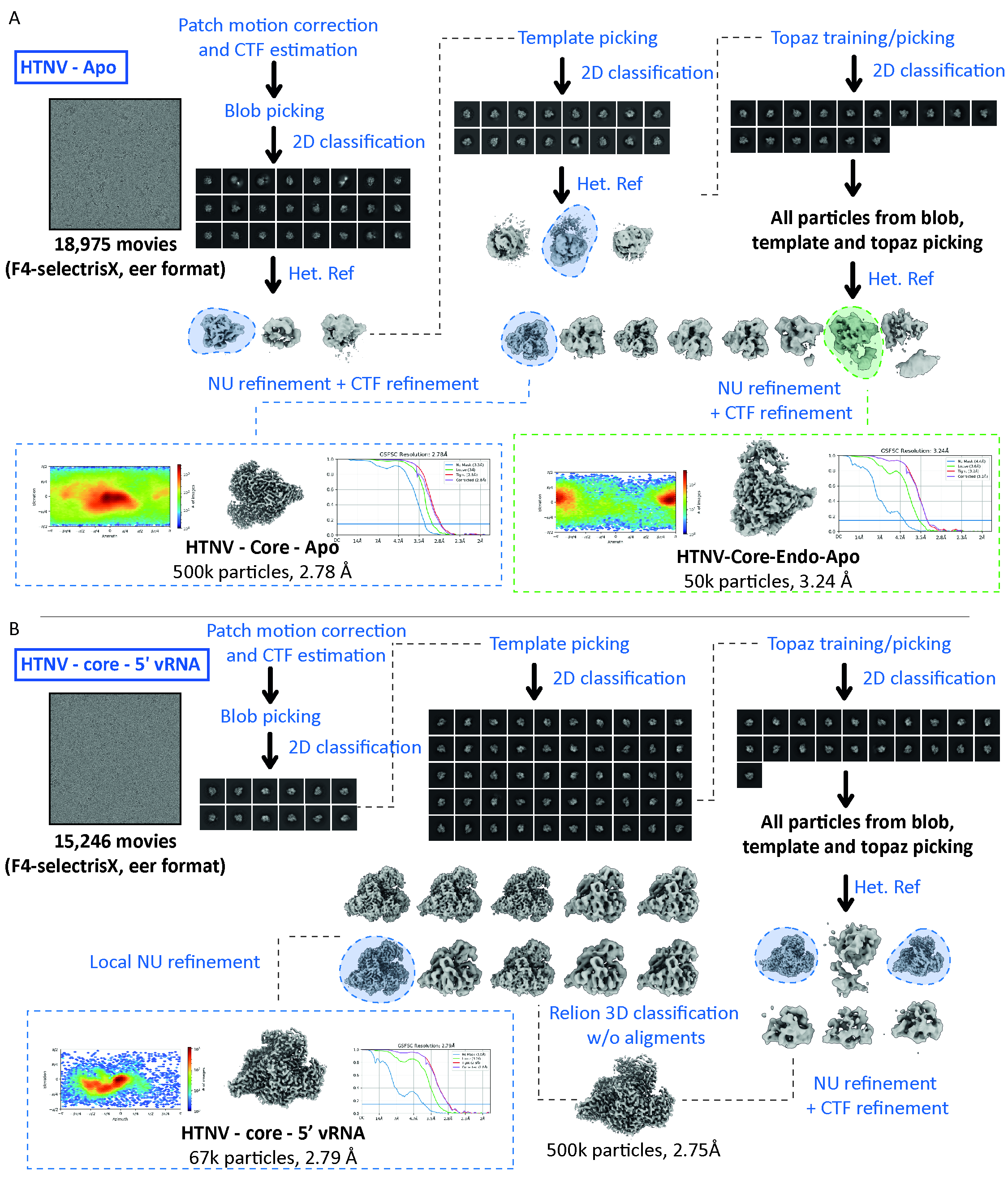

Supplement: S2 Fig — Processing schemes for the RNA free (A) and 5′ RNA only (B) HTNV cryo-EM datasets. (TIF) [file ppat.1012781.s003.tif]

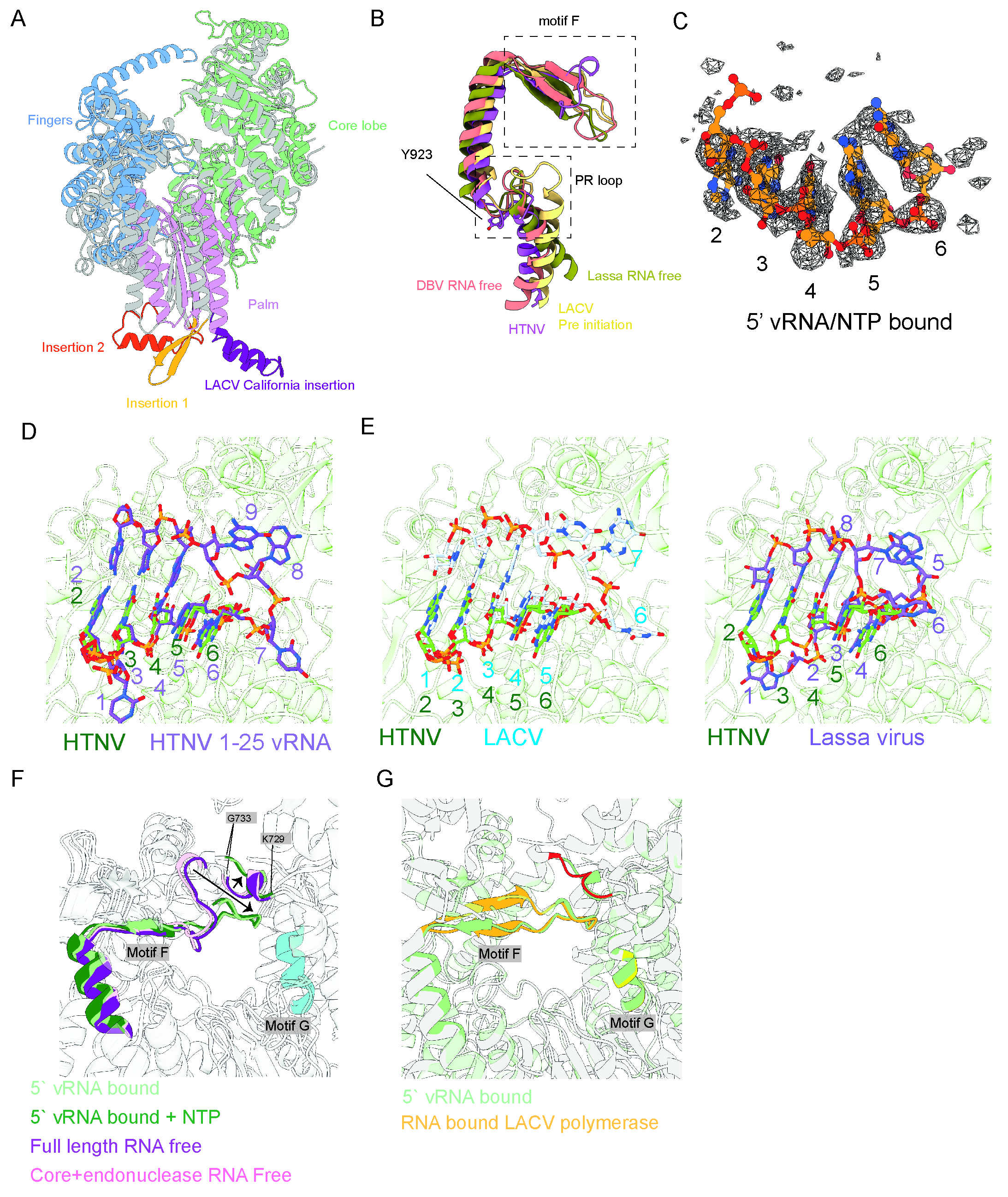

Supplement: S3 Fig — (A) Insertions into the palm domain from HTNV (grey) with insertions 1 (yellow) and insertion 2 (red) annotated. The structure of the LACV polymerase (PDBID: 6Z6G) (blue, green, pink) are shown with the California like insertion (purple). (B) The position of the prime-realign (PR) loop is shown in the retracted position from HTNV, DBV (PDBID: 6Y6K), LACV pre-initiation state (PDBID: 6Z6G), and RNA free Lassa virus (PDBID: 6KLC) polymerase are shown. The location of the PR loop and motif F is annotated. (C) Electron density for the bases observed in the 5′ hook structure from the 5′ vRNA bound + NTP model. (D) The HTNV vRNA binding site from the 5′ vRNA for HTNV (green) is compared to the 1–25 vRNA bound HTNV model (purple). (E) The alignment of the register of the 5′ vRNA from HTNV (green), LACV (blue, PDBID: 7ORK) and lassa virus (purple, PDBID: 7OJL) are shown with base numbers annotated. (F) The position of motif F changes in response to the binding of the 5′ vRNA promoter. (G) LACV polymerase (PDBID: 7ORK), when the 5′ vRNA promoter is bound, motif F is similarly arranged to that observed in HTNV. HTNV polymerase (green) and the LACV (yellow, red) are shown. (TIF) [file ppat.1012781.s004.tif]

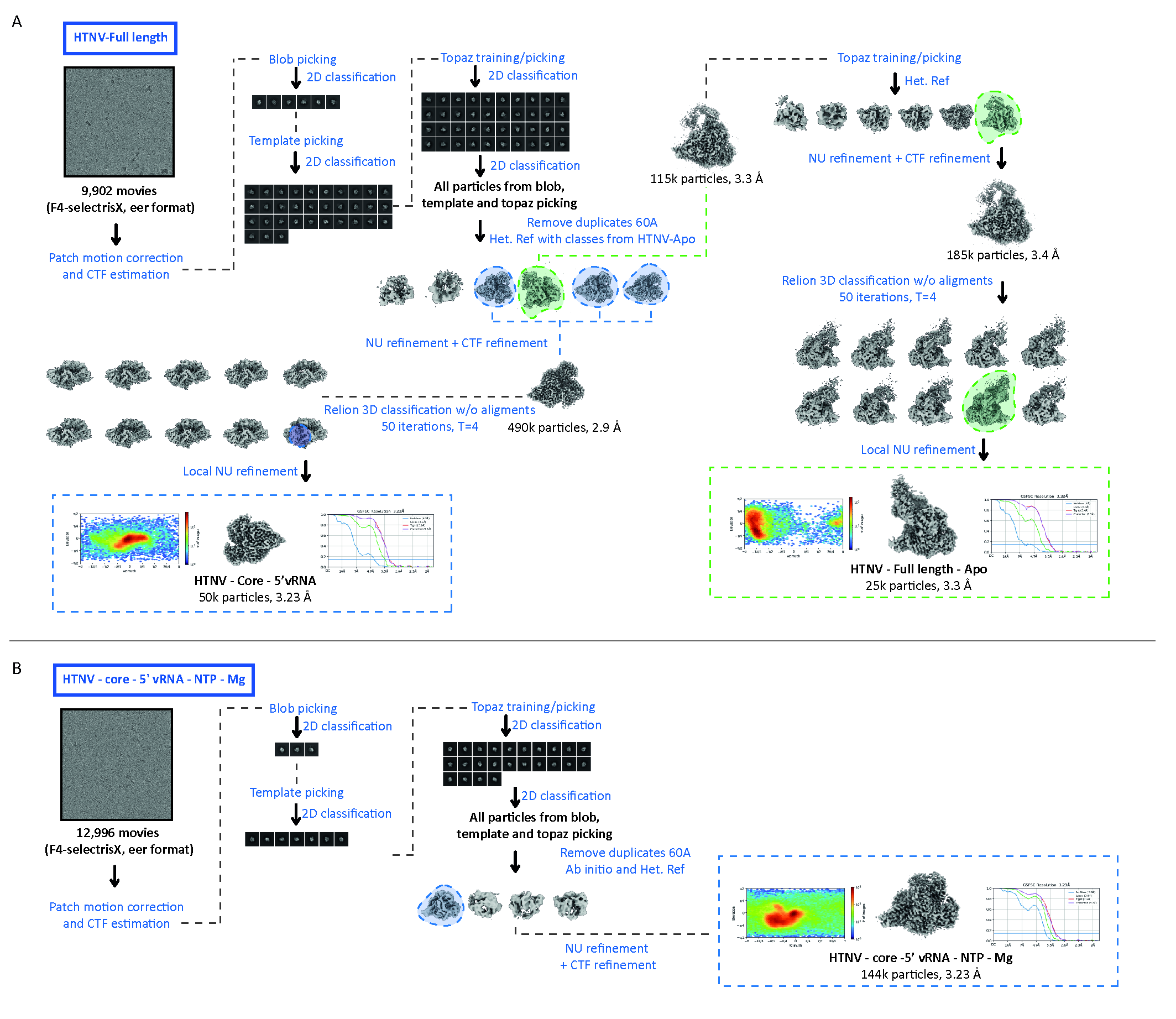

Supplement: S4 Fig — Processing schemes for the Full-length (A) and RNA and NTP (B) bound HTNV cryo-EM datasets. (TIF) [file ppat.1012781.s005.tif]

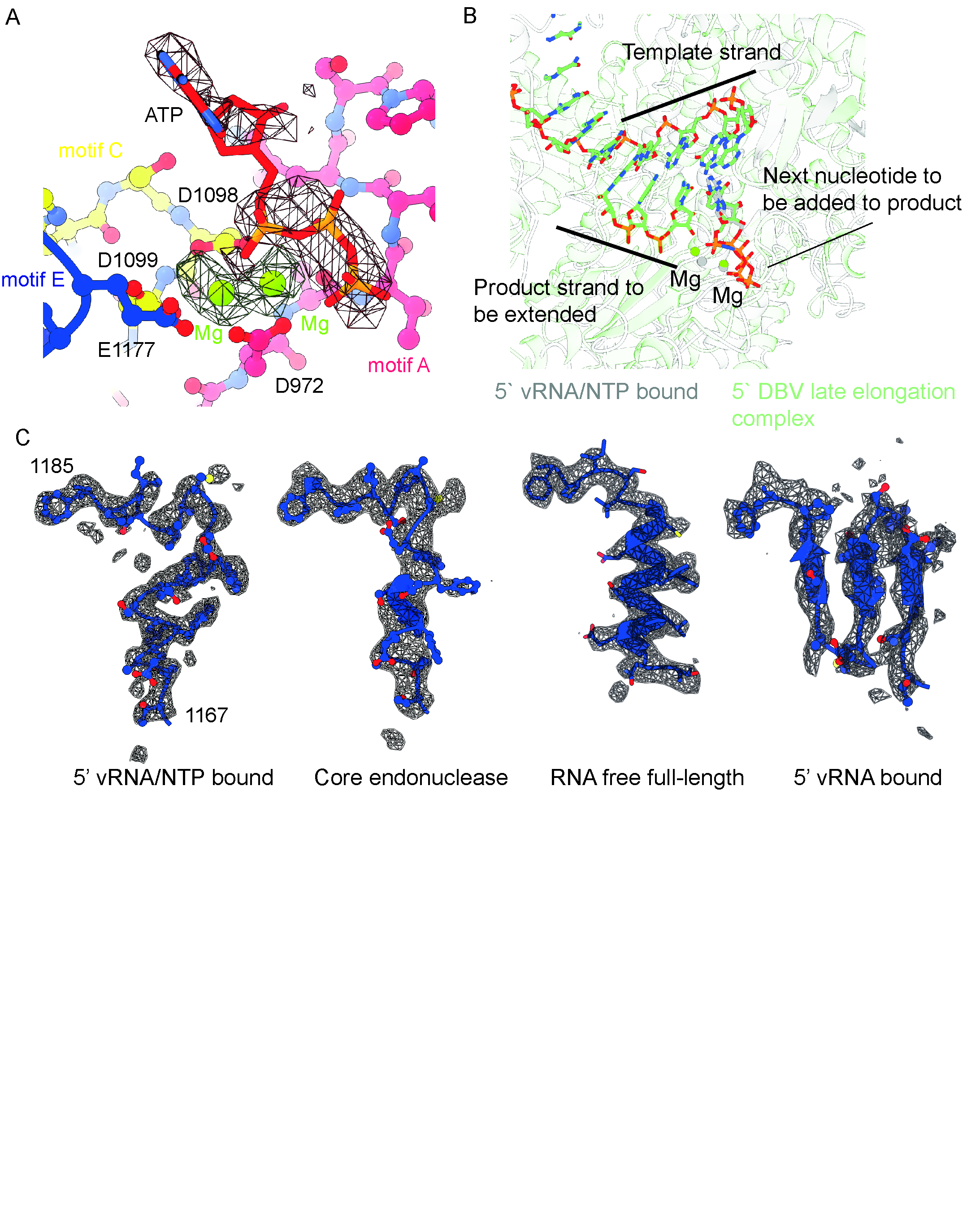

Supplement: S5 Fig — (A) Density map for the nucleotide in the polymerase active site with interactions residues annotated. (B) HTNV (grey) and DBV (green) (PDBID: 8ASD) polymerase are overlaid showing the conservation of the nucleotide binding site in the polymerase active site. The product and template RNA strands from DBV polymerase are shown in stick representation. (C) Density map for residues 1167–1185 which undergo large rearrangement. (TIF) [file ppat.1012781.s006.tif]

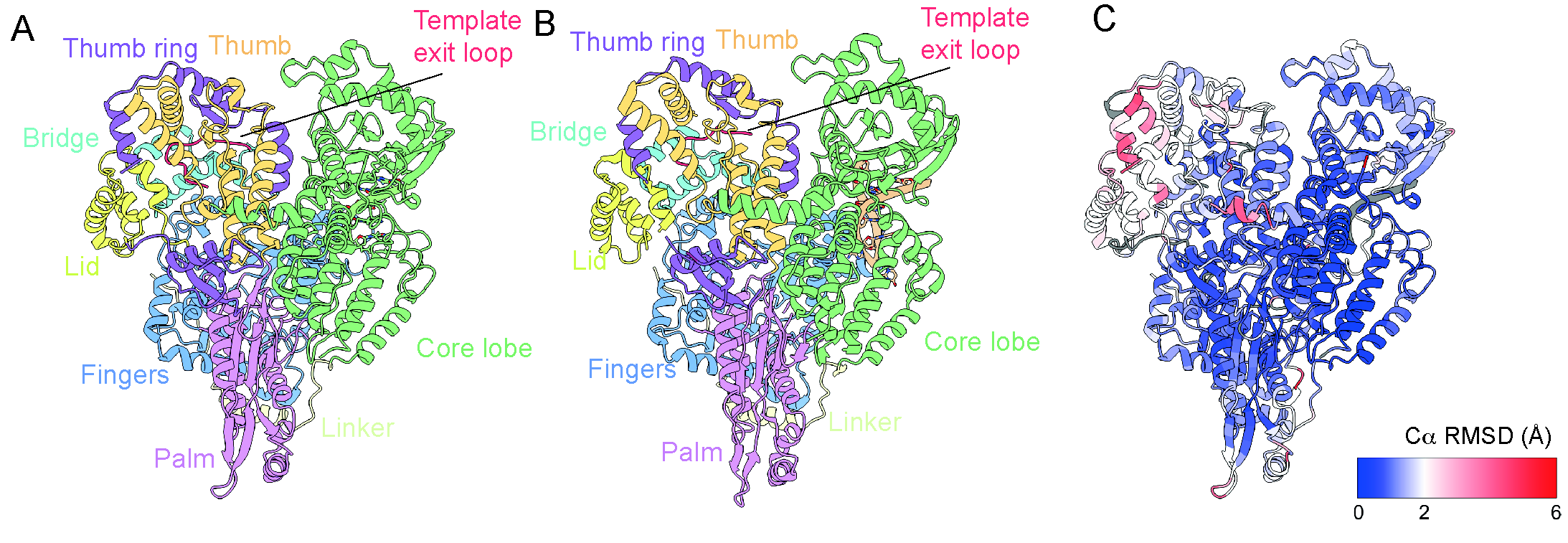

Supplement: S6 Fig — Comparison of HTNV (PDBID: 8P1N) (A) and Sin Nombre (PDBID:8CI5) (B) core with 5’ promoter RNA bound with domains annotated. (C) The HTNV structure has been coloured according to the RMSD of equivalent Ca. Global RMSD of equivalent (1022 residues) Ca is 1.03 Å. (TIF) [file ppat.1012781.s007.tif]
