# Supplementary material for: Structural characterization of the full-length Hantaan virus polymerase
Source: PLoS Pathog. 2024 Dec 9;20(12):e1012781. doi: 10.1371/journal.ppat.1012781 (PMC11658695; doi:10.1371/journal.ppat.1012781)
Supplement: Attachment — Submitted filename: Keown_HTNV_Reviewer_comments_response.docx [file ppat.1012781.s008.docx]

**Part I - Summary**

Reviewer #1: The manuscript by Keown et al. describes several structures of the HTNV polymerase including protein constructs of two different lengths and with and without bound RNA. While significant structural work on HTNV polymerase has been previously published, this work details several new findings including conformations of motif F that block template binding and a full-length structure of the polymerase. Despite enthusiasm for these novelties, there are several concerns primarily relating to the interpretation of the authors’ EM maps. While the findings are interesting both the content and the descriptions in the text should be rigorously reevaluated.

We thank the reviewer for their positive assessment of our work and detail below our changes to their concerns.

**Part II – Major Issues: Key Experiments Required for Acceptance**

Reviewer #1: Line 260: The identification of a nucleotide in the active site is unjustified. Even taking the map threshold very low, it is not possible to distinguish any part of the nucleotide or magnesium ions from the noise. It isn’t clear how the authors produced figure S6A, but I cannot reproduce it with their maps.

Please double click to activate movie

Figure 1. Showing the NTP coordination in the active site.

We appreciate the reviewer for spending the effort to assess the important molecular details of our maps and models. To help demonstrate the quality of the density for the NTP we have downloaded the PDB 8P1M and EMD 17354 directly from RCSB. These have then been opened in chimerax, the threshold set to 0.191, the NTP centred, and the above movie recorded. We consider the sidechains density (3Asp and 1Glu at the bottom of the image), the two mg ions (green spheres), and NTP to be sufficiently well ordered to model in our map. We have included a static image prepared at the same map threshold and annotated with the important features.

To prepare Fig S6A (now S5A) we performed the following steps. First map threshold was set to 0.216. next, molecular features, including NTP, MG, and protein motifs were coloured. From these colouring the map was then split (based on model colour) so we could specifically show the density for the Mg and NTP and key residues without the confusing background from the surrounding map region. We believe the features of the map, as represented in Fig S5A accurately represent the strength of the density present in the original map.

To further support the positioning of the NTP and Mg ions, we note in the related SFTSV structural model (PDB ID: 8ASD) both NTP and Mg are observed at very similar locations.

Fig S7: Given the weak nature of the bands and large number of alternate products. I would prefer to see replicates of the ApG extensions with polymerase mutants.

We have now repeated these assays several times, under several related conditions. Our replicates (below Fig 2) also revealed a complex pattern of bands; furthermore, the 5’ alone has been shown (below Fig 3) to result in a range of products that further complicates the analysis.

Reassessing the data, although we remain convinced that our original conclusion is correct, based in presence of bands unique to the D97A mutant, and the lack of these bands for the other mutants, nevertheless we have decided to omit these data to avoid potential misinterpretation by the reader. Clearly, this is something that needs further exploration. We are grateful to the reviewer for highlighting this and this is an ongoing focus of our continuing project.

Fig2. Replicate Gels 1 and 2 similar to the original gel in the submitted manuscript.

Fig 3. Gel showing strong extension in the presence of 5’ or 3’ vRNA. Reactions are either denovo primed, UpA, or ApG primed.

**Part III – Minor Issues: Editorial and Data Presentation Modifications**
Please use this section for editorial suggestions as well as relatively minor modifications of existing data that would enhance clarity.

Reviewer #1: Line 133: Claims of an ordered endonuclease domain in 8P1K seem overstated. The resolution of this domain is significantly lower than the core portion of the structure and much of the domain is entirely absent at low threshold.

While we agree that the endonuclease is less well-ordered than the polymerase core we believe our modelling is appropriate supported. During our initial PDB deposition we have uploaded this map an additional map. Above we have prepared a figure showing the fit of the endonuclease into this map. The model which we have used here is not built ab initio, instead hybrid modelling using Alphafold2 and an existing structure from the PDB 5IZE (Hantaan endonuclease structure). These models were fit with only minor adjustments to maintain connectivity to the core. Please see the above figure which we believe supports our positioning of the domain.

We have added a more accurate description of our modelling approach to our methods section (lines 554-563) and described our use of deepEMhancer to generate more interpretable maps (lines 546-549).

Line 137: Not clear what the reference to “261-273” is referring to as it appears to not be the endonuclease, core lobe or linker.

We apologise for the typo error here and have revised this to 215-261 which now accurately reflects our domain boundaries in Fig.1a

Line 158: the observation of insertions in the palm domain appear to make a claim to novelty, but these structures were previously observed in Durieux et al. 2023.

To reflect that these regions were observed in work which was submitted around the same time as our study we have added the statement “ … as reported in recent similar studies [23,24]”.

Line 189: Not clear what “different” piece of 5’ vRNA the authors are referring to here. 8QH3 appears to have an identical RNA sequence bound at this position.

We apologise for the confusion. In our study we observe a short 5 base fragment of the 5’ vRNA, while in the 8QH3 model, they observe 11 bases. The observed region in our model (5′-UAGUAGUAGUAUGCUCC-3′) matches a section of the observed fragment in the 8QH3 (5′- UAGUAGUAGACACCGCAAGAUGUUA-3′) where they see additional bases. Red colouring shows identical bases observed in both models, orange shows additional bases in 8QH3, and black unobserved bases.

We have amended the text “… those recently reported with an extended section of 5′ vRNA [23].”

Line 218: The C-terminal domain, while clearly present in the map, is largely disordered. While the domain as modelled is one possible interpretation of this map, structural homology searches and comparisons mentioned in the text are somewhat dubious.

We believe there are two different points raised by the reviewer here and will address these separately. Our modelling of this domain was initially based on the high confidence AF2 modelling of the core of this region and has subsequently been validated again experimentally by the structure in PMID: 38480734. For these reasons we our comparisons are valid.

We agree with the reviewer that the C-terminal domain is in a region of weak electron density. Our modelling of the domain is limited to a rigid-body fit of the region is supported from the observations in AF2 and from additional experimental data PMID: 38480734.

To further support our initial positioning of the domain we have attempted to resolve this region to higher resolution using the Relion-Blush algorithm. We did not observe any additional improvement. We have additionally used deepEMhancer and alternate bfactor sharpening to increase the interpretability of the region. In the below figure we have shown has both approaches independently support the positioning of this region. To aid in the assessment we have highlighted four helices which we believe are unambiguously positioned. We have contacted the PDB to update our depositions with these additional maps.

Line 229: While there is clearly a shift in the endonuclease domain between 8P1K and 8P1L, the poor density for the endonuclease in map 17352 precludes any sort of conclusion about changes in this regions conformation or contacts as seen in the full-length structure.

We have removed this figure panel from the manuscript.

Line 232: The authors have actually built three conformations for this loop. The density for this region in 8P1M/17354 is poor though and it isn’t sure what the justification for an alternate build here is.

We have updated the Mg.NTP bound model as suggested by the reviewer, and have removed residues 1318-1329 given the weakness of the density. We have updated Fig3A inset and contacted the PDB to have 8P1M updated. Minor changes to the main text now remove the discussion of the NTP bound structure being different in this region.

Line 310: Presumably “the position” of motif E being referred to is the alpha helical conformation?

We have updated the text, removing “position” to “helical arrangement” to be specific about which secondary structure would affect transcription.

Line 325: I am not convinced that the authors are seeing four distinct conformations of motif E. 8P1J, 8P1K and 8P1M all have the same short helix, 8P1L contains the longer helix and 8P1N contains the beta-sheet. It isn’t clear here what the authors are referring to as the “ordered loop” arrangement.

We apologise for the confusion here. In our analysis consider the two Apo models (8P1J and 8P1K) to be the same but 8P1M as a different model which we initially termed as the “ordered loop” conformation. We agree that the N-terminal region of motif E (approximately residues 1169-1174) are in a similar short helix and similarly positioned. The difference between these models comes in residues 1175-1183 which contain the key residue E1177 which we propose coordinates an active site Mg. In the Apo and Mg.ATP models 1175-1183 undergo substantial rearrangement which causes a repositioning of E1177.

To clarify the role of this loop we have removed references to the “ordered loop” and it is now called the nucleotide primed conformation, recognising this conformation’s role in coordination of Mg and nucleotide.

Line 570: Update to indicate 8P1L pairing with EMDB-17353. 8P1M appears to be 5’ vRNA + ATP and 8P1N appears to bound to just 5’ vRNA. Database entries appear to be correct.

We have corrected this error.

Fig 3A, 4ACD: Please avoid using yellow text, it is very hard to read.

We have updated these areas with grey boxes behind the yellow text to improve clarity.

Fig S2, S4: Figures and text need to be at a size that they can be read

We have updated these two figures with increased text and image sizes.

Figure Files:

While revising your submission, please upload your figure files to the Preflight Analysis and Conversion Engine (PACE) digital diagnostic tool, [https://pacev2.apexcovantage.com](https://pacev2.apexcovantage.com/). PACE helps ensure that figures meet PLOS requirements. To use PACE, you must first register as a user. Then, login and navigate to the UPLOAD tab, where you will find detailed instructions on how to use the tool. If you encounter any issues or have any questions when using PACE, please email us at [figures@plos.org](mailto:figures@plos.org).
