# Supplementary material for: Structural characterization of the full-length Hantaan virus polymerase
Source: PLoS Pathog. 2024 Dec 9;20(12):e1012781. doi: 10.1371/journal.ppat.1012781 (PMC11658695; doi:10.1371/journal.ppat.1012781)
Supplement: Attachment — Submitted filename: Response_to_revision_2.docx [file ppat.1012781.s009.docx]

Dear Dr. Grimes,

Thank you for submitting your manuscript to PLOS Pathogens. After careful consideration, we feel that it has merit but does not fully meet PLOS Pathogens's publication criteria as it currently stands. Therefore, we invite you to submit a revised version of the manuscript that addresses the points raised during the review process.

Please submit your revised manuscript within 60 days Jan 19 2025 11:59PM. If you will need more time than this to complete your revisions, please reply to this message or contact the journal office at plospathogens@plos.org. When you're ready to submit your revision, log on to https://www.editorialmanager.com/ppathogens/ and select the 'Submissions Needing Revision' folder to locate your manuscript file.

Please include the following items when submitting your revised manuscript:

* A rebuttal letter that responds to each point raised by the editor and reviewer(s). You should upload this letter as a separate file labeled 'Response to Reviewers'. This file does not need to include responses to any formatting updates and technical items listed in the 'Journal Requirements' section below.

* A marked-up copy of your manuscript that highlights changes made to the original version. You should upload this as a separate file labeled 'Revised Manuscript with Track Changes'.

* An unmarked version of your revised paper without tracked changes. You should upload this as a separate file labeled 'Manuscript'.

If you would like to make changes to your financial disclosure, competing interests statement, or data availability statement, please make these updates within the submission form at the time of resubmission. Guidelines for resubmitting your figure files are available below the reviewer comments at the end of this letter.

We look forward to receiving your revised manuscript.

**Journal Requirements:**

Please amend your detailed Financial Disclosure statement. This is published with the article. It must therefore be completed in full sentences and contain the exact wording you wish to be published.

Please ensure that the funders and grant numbers match between the Financial Disclosure field and the Funding Information tab in your submission form. Note that the funders must be provided in the same order in both places as well.

We thank the reviewers/editors for the positive assessment of our work.

We have now revised our manuscript submission and manuscript to include the below financial disclosure statement in line with journal standards.

This work was supported by Wellcome Investigator Awards (200835/Z/16/Z and 222510/Z/21/Z to JMG), a University of Oxford-Medical Sciences Division Pump Priming Award (0012329 to JRK), a Medical Research Council programme grant (MR/R009945/1 to E.F.), the Deutsche Forschungsgemeinschaft (DFG, German Research Foundation) under Germany's Excellence Strategy - EXC 2155 (390874280 to BEN) and the Swedish Research Council (2023-02595 to BEN). Access to electron microscopes was provided by the OPIC Electron Microscopy Facility (funded by Wellcome JIF (060208/Z/00/Z) and equipment (093305/Z/10/Z) grants). Access to computational resources was supported by a Wellcome Trust Core Award Grant (203141/Z/16/Z. The views expressed are those of the author(s) and not necessarily those of the NHS, the NIHR, or the Department of Health. The funders had no role in study design, data collection and analysis, decision to publish, or preparation of the manuscript.

s
